# Lipopolysaccharides Modulate Cellular Responses in Dental Pulp Cells

**DOI:** 10.1155/ijod/2049490

**Published:** 2026-04-23

**Authors:** Tanida Srisuwan, Chatvadee Kornsuthisopon, Nunthawan Nowwarote, Xiaofei Zhu, Waruna Lakmal Dissanayaka, Thanaphum Osathanon

**Affiliations:** ^1^ Department of Restorative Dentistry and Periodontology, Faculty of Dentistry, Chiang Mai University, Chiang Mai, Thailand, cmu.ac.th; ^2^ Centre of Excellence for Dental Stem Cell Biology and Department of Anatomy, Faculty of Dentistry, Chulalongkorn University, Bangkok, Thailand, chula.ac.th; ^3^ INSERM UMR1163, Imagine Institute, Université Paris Cité, Paris, France, u-paris.fr; ^4^ Department of Oral Biology, Faculty of Dentistry, Université Paris Cité, Paris, France, u-paris.fr; ^5^ Department of Endodontics, Henry M. Goldman School of Dental Medicine, Boston University, Boston, USA, bu.edu; ^6^ Applied Oral Sciences and Community Dental Care, Faculty of Dentistry, The University of Hong Kong, Hong Kong, Hong Kong SAR, China, hku.hk

**Keywords:** dental pulp, differentiation, inflammation, lipopolysaccharide, oxidative stress

## Abstract

Lipopolysaccharide (LPS) triggers signal transduction in dental pulp cells (DPCs), leading to various biological events. The present study described the biological response of DPCs to LPS, aiming to provide a deeper understanding of its mechanisms. A literature search was performed in PubMed, Web of Science and Google Scholar using the keywords ‘lipopolysaccharide’, ‘dental pulp cells’ and ‘dental pulp stem cells’. Original studies and review articles published in English that investigated cellular or molecular responses of pulp‐derived cells to LPS stimulation were included. A narrative review was then conducted to summarise and discuss the effects of LPS on these cells. Across the selected studies, LPS exposure consistently activates intracellular signalling cascades in DPCs, leading to a series of downstream events, including inflammatory cytokine production, oxidative stress generation, mitochondrial impairment, premature cellular senescence and alterations in odonto/osteogenic differentiation. These interconnected molecular changes underpin the progression of pulpal inflammation and influence the tissue’s capacity for repair or regeneration. This narrative review described the biological response of DPCs to LPS, providing deeper insight into its mechanisms. Understanding LPS‐mediated signalling enables clinicians to tailor regenerative therapeutic approaches and vital pulp therapies by modulating inflammation and optimising biological events to support dental pulp tissue healing and dentine regeneration.

## 1. Introduction

The dental pulp is a richly vascularised and innervated soft connective tissue enclosed by mineralised tissues [1]. It plays a vital role in maintaining tooth vitality by providing essential nutrients, sensory function, and contributing to defence mechanisms [1]. The pulp comprises various cell types, including odontoblasts, fibroblasts, endothelial cells, immune cells, and a population of mesenchymal stem cells (MSCs) known as dental pulp stem cells (DPSCs) [2]. These cells contribute not only to routine tissue maintenance but also to reparative and regenerative responses following injury.

Pulpal inflammation, or pulpitis, typically arises from bacterial invasion but can also result from trauma, restorative procedures, or chemical irritants [1, 3]. Among the microbial agents, Gram‐negative bacteria are of particular significance due to their outer membrane component, lipopolysaccharide (LPS), which serves as a potent pro‐inflammatory molecule [4]. LPS is recognised directly by DPSCs, triggering intracellular signalling that rapidly induces the release of key inflammatory mediators, including interleukin (IL)‐1β, IL‐6, and tumour necrosis factor‐α (TNF‐α) [5]. While this acute inflammatory response is essential for microbial clearance and dentine/pulp tissue healing, sustained or excessive inflammation can lead to irreversible pulp damage and subsequently necrosis [1].

Over the past decade, there has been growing interest in understanding how LPS influences the biological behaviour of dental pulp cells (DPCs) and DPSCs [6, 7]. The terminology used for dental pulp–derived cells varies widely across studies. In some reports, ’DPCs’ refers to mixed primary pulp cells, while others use the same term for stem‐like populations. Likewise, ’DPSCs’ is generally used to refer to stem/progenitor cells, although this definition is not consistently applied in the literature. DPCs exhibit greater diversity, as they encompass various mesenchymal cell types from dental pulp tissues, whereas DPSCs are isolated explicitly from dental pulp and possess MSC characteristics. In this review, DPCs refer to heterogeneous primary pulp cells, whereas DPSCs denote stem/progenitor cells with mesenchymal characteristics. These cells not only respond to inflammatory stimuli but also actively participate in modulating the immune response. A previous study revealed that DPSCs express Toll‐like receptors (TLRs) 1–10 under a non‐inflammatory environment, though at varying levels [8]. Notably, under inflammatory stimulation, the expression of TLRs 2, 3, 4, 5, and 8 increases, while TLRs 1, 7, 9, and 10 decrease, and TLR6 may even be completely suppressed [8]. These shifts reflect a selective adjustment of innate immune signalling in the inflamed pulpal environment. Emerging evidence suggests that the nature and magnitude of LPS exposure, whether transient or consistent, can dictate cellular outcomes ranging from enhanced reparative activity to cellular senescence. Furthermore, the immunomodulatory properties of these cells under inflammatory conditions have significant implications for regenerative dentistry. A more precise understanding of how DPCs and DPSCs respond to LPS across different doses and exposure durations can help explain why pulp inflammation progresses differently among individuals. Summarising these cellular and molecular responses also provides a foundation for identifying biological targets that may support pulp preservation and regeneration.

## 2. Signalling Pathways Activated by LPS

LPS is a well‐characterised component of the outer membrane of Gram‐negative bacteria that plays a central role in the pathogenesis of pulpitis. Structurally, LPS is composed of three distinct domains: lipid A, the core oligosaccharide, and the O‐antigen (Figure S1). Of these, lipid A is primarily responsible for the molecule’s potent endotoxic activity [9]. In the context of dental infections, LPS derived from various species such as *Escherichia coli*, *Porphyromonas gingivalis*, *Fusobacterium nucleatum*, and *Prevotella intermedia* is frequently utilised as a virulent factor in the experiments, as these bacterial species can be detected in carious lesions and infected pulp tissue [10, 11]. Typically, LPS is isolated from bacterial cell walls using phenol‐water extraction or enzymatic digestion and is often obtained in a purified, standardised form from commercial sources to ensure experimental consistency.

Although LPS plays a critical role in initiating inflammation, the detailed molecular mechanisms underlying its interaction with cells, particularly DPCs and DPSCs, remain unclear. One key mechanism underlying LPS‐induced responses involves its recognition by the innate immune system, particularly through the lipid A component of LPS, which binds to myeloid differentiation factor 2 (MD‐2) and forms a complex with TLRs [12]. CD14 functions as a co‐receptor in the detection of LPS, collaborating with TLR4 and MD‐2. It plays a crucial role in identifying LPS and triggering the immune response. In parallel, the Wnt5a signalling pathway has also been implicated in LPS‐related inflammatory responses [13]. Aside from this, LPS may influence gene expression by altering the DNA methylation profile, regulating noncoding RNA particles, and modulating inflammation through microRNA‐related mechanisms [14–16].

**Figure 1:**
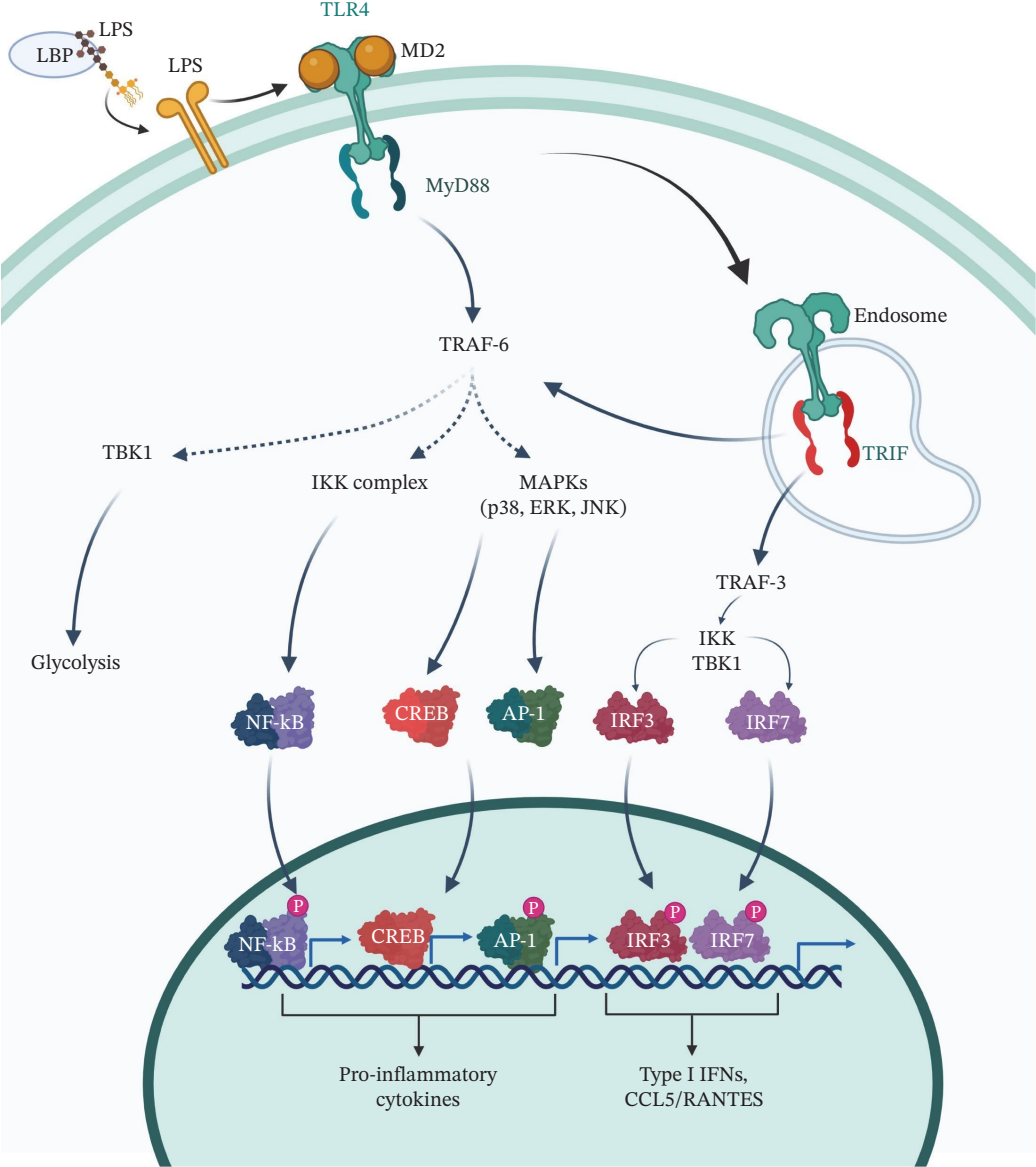
TLR4 signalling cascade. TLR4 is the primary receptor responsible for LPS binding. Upon ligand binding, TLR4 activates the MyD88 signalling pathway at the plasma membrane, initiating several intracellular signalling cascades, including the IKK complex and MAPKs, which in turn promote the production of pro‐inflammatory cytokines. The activation of TBK1 pathway lead to glycolysis. In addition, the TRIF pathway can also be activated, resulting in the transcription of type I interferon and CCL5/ RANTES (modified from [18]). Created in BioRender https://BioRender.com/gk759tc.

In the primary pathway, LPS functions as a PAMP recognised by PRRs expressed on various pulp‐resident cells, including odontoblasts, fibroblasts, immune cells, and dental stem cells [12]. There are five categories of PRRs, namely: TLRs, nucleotide oligomerisation domain (NOD)‐like receptor (NLRs), retinoic acid‐inducible gene‐I (RIG‐I)‐like receptors (RLRs), C‐type lectin receptors (CLRs), and absent in melanoma‐2 (AIM2)‐like receptors (ALRs) [17]. Among these PRRs, TLRs are the most studied, particularly TLR4, which plays a central role in recognising LPS (Figure 1). TLRs are type I transmembrane proteins consisting of extracellular leucine‐rich repeats (LRRs), a single transmembrane domain, and a cytoplasmic Toll/IL‐1 receptor (TIR) domain. Of the 10 TLRs identified in MSCs, TLR4 is the primary receptor responsive to LPS binding [19]. Upon ligand engagement, the TIR domain mediates the recruitment of adaptor molecules such as MD‐2 to initiate downstream signalling cascades [19, 20]. Since MD‐2 lacks a transmembrane domain, it remains anchored to the cell membrane through its interaction with TLR4, forming a functional receptor complex [21]. A recent study confirmed that 20 µg/mL of LPS significantly upregulated TLR4 and MD‐2 expression at both the mRNA and protein levels [22]. Pretreatment with MD‐2 inhibitors, including MAC28, L6H21, or 2i‐10 (10 µM) for 2 h before LPS exposure, significantly reduced the expression of TLR4, MD‐2, TNF‐α, and IL‐6, demonstrating their efficacy in suppressing TLR4‐MD‐2‐mediated inflammation. These findings demonstrated that the TLR4–MD–2 signalling pathway is a key mechanism of LPS‐induced inflammation in heterogeneous human primary pulp cells (hDPCs) [22]. However, it should be emphasised that the structure of the lipid A portion of LPS directly affects its binding affinity to MD‐2 and the efficiency of TLR4‐MD‐2 dimerisation. These structural variations are crucial determinants of the strength and outcome of the inflammatory signalling triggered by LPS [23].

Following ligand recognition, TLR4 activation initiates multiple downstream signalling cascades that vary depending on the cell type involved. One major pathway is the MyD88‐dependent cascade, which leads to the activation of mitogen‐activated protein kinases (MAPKs), such as ERK, JNK, and p38. Additional pathways include type I interferon‐dependent MAPK signalling, TRIF (TIR domain–containing adapter‐inducing interferon‐β)‐mediated responses, and the PI3K‐Akt signalling axis [18, 24–26]. Together, these signalling routes regulate key cellular processes including proliferation, migration, and differentiation. They also contribute to the upregulation of pro‐inflammatory mediators such as TNF‐α, IL‐6, and IL‐8, as well as matrix‐degrading enzymes, such as matrix metalloproteinases (MMPs), which further promote tissue remodelling and inflammation [18, 27, 28]. A recent clinical investigation of isolated human dental pulp tissues from teeth with irreversible pulpitis demonstrated a marked increase in phosphorylated NF‐κB levels, indicating activation of the NF‐κB‐dependent inflammatory signalling pathway [29]. In contrast, a study by the same group evaluated phosphorylated NF‐κB expression in DPC lysates after LPS induction. They reported that LPS did not alter NF‐κB phosphorylation levels, suggesting possible compartment‐specific NF‐κB signalling activation (e.g., nuclear vs cytosolic) [30].

Another signalling axis involved in LPS‐induced responses is Wnt5a, a non‐canonical Wnt family member that regulates stem cell differentiation and modulates inflammatory responses in immune cells. Wnt5a expression has been shown to be upregulated via TLR4/MyD88, PI3K/Akt, and NF‐κB signalling pathways, highlighting its significance in the context of pulpal inflammation [13]. In addition, some studies have reported alterations in transcription factors following LPS stimulation. For example, TET2, a DNA methylcytosine dioxygenase, is upregulated and promotes hydroxymethylation of genes such as MyD88, enhancing their expression [14]. LPS also induces OCT4B1 expression, leading to increased OCT4B levels [31]. Moreover, LPS has been shown to downregulate the mRNA levels of DNA methyltransferase 3B (DNMT3B) and both the mRNA and protein levels of DNA methyltransferase 1 (DNMT1) [32]. Deregulation of these DNA methyltransferases leads to elevated proinflammatory mediators and altered microRNA expression profiles.

## 3. Cellular Effects of LPS

Following TLR4 activation by LPS, cells undergo a cascade of intracellular changes that significantly alter their physiological functions. These cellular responses extend beyond classical inflammation and involve intricate modulation of redox balance, mitochondrial dynamics, stem cell senescence and differentiation potential. The following sections examine the diverse cellular effects of LPS, with a focus on both heterogeneous and stem cell populations of dental pulp‐derived cells, DPCs and DPSCs, particularly in relation to inflammatory signalling, oxidative stress, mitochondrial function, cellular senescence and odontogenic differentiation. Although various studies have explored LPS exposure across different experimental contexts, the precise concentrations and durations required to elicit specific cellular responses remain uncertain. The following sections focus on and discuss these aspects. To improve clarity, the present review defines ‘mild stimulation’ as concentrations typically ≤1 µg/mL and ’high‐dose exposure’ as concentrations ≥10–20 µg/mL, based on commonly used experimental ranges reported in the included studies. In addition, LPS exposure durations are broadly categorised as short‐term (≤24 h), which typically elicits acute inflammatory or oxidative responses, and prolonged exposure (≥48 h to several days), which is more commonly associated with mitochondrial dysfunction, senescence and impaired differentiation. These ranges are intended as approximate reference points, acknowledging that cellular responses may vary across studies due to differences in cell source, experimental duration and LPS origin.

### 3.1. Inflammatory Effects of LPS on DPCs and DPSCs

Dental pulp tissue serves as the primary site of response to bacterial invasion. Upon stimulation with LPS, resident cells within the dental pulp undergo phenotypic and molecular alterations that initiate and sustain the inflammatory cascade associated with pulpitis.

#### 3.1.1. Cytokine and Chemokine Production

One hallmark response of both heterogeneous and stem cell populations of dental pulp‐derived cells, DPCs and DPSCs, to LPS stimulation is the robust secretion of pro‐inflammatory cytokines and chemokines. *P. gingivalis* LPS (10 µg/mL) promoted IL6, IL8 and IL12 expression in DPCs [33, 34]. Acting primarily through the TLR4/CD14/MD‐2 complex, LPS activates downstream signalling pathways such as NF‐κB and MAPKs, resulting in elevated expression and secretion of key inflammatory mediators, including IL‐1β, IL‐6, IL‐8 and TNF‐α [6, 35, 36]. These cytokines orchestrate the recruitment and activation of immune cells, such as neutrophils and macrophages, into the pulp, setting the stage for both pathogen elimination and collateral tissue damage. Bindal et al. [37] concluded that treating human DPSCs (hDPSCs) with 1 µg/mL of *E. coli* LPS for 24 h is an optimal approach to induce an inflammatory microenvironment, as indicated by increased levels of IL‐6, IL‐8, tissue plasminogen activator (tPA), and tachykinin precursor 1 (TAC1) [37]. Although LPS alone did not immediately enhance IL‐6 production during the early phase (within 48 h), prolonged exposure over 7 days resulted in a significant, concentration‐dependent increase in IL‐6 secretion [38]. Similarly, various studies have reported that higher concentrations of LPS, for example, 20 µg/mL, cause a significant increase in IL‐6 and TNF‐α expression in DPCs compared to lower concentrations, confirming pro‐inflammatory activation [22, 30, 39]. Additionally, IL‐1β levels rose proportionally with LPS concentrations, suggesting a progressive and sustained inflammatory response [38]. Unregulated overexpression of IL‐1β may play a critical role in intensifying inflammation and potentially contributing to pulp tissue necrosis [40]. It has been shown that blocking IL‐1 signalling with IL‐1RA significantly reduced both IL‐1β and TNF‐α at gene and protein levels, without substantially affecting anti‐inflammatory cytokines such as IL‐10 in co‐culture of LPS‐activated hDPSCs and macrophages, highlighting the critical role of IL‐1β in sustaining pulpal inflammation [41].

Interestingly, despite the absence of corresponding gene expression, TNF‐α levels were significantly elevated, which may be attributed to LPS activating inflammation via dual receptor pathways, TLR4 and TLR2 [37]. The innate immune response, particularly involving macrophages, appears to be influenced by DPSCs through the TNF‐α/indoleamine 2,3‐dioxygenase (IDO) regulatory axis [40]. Activation of TLR4 by LPS leads to NF‐κB p65 subunit translocation into the nucleus and subsequent expression of pro‐inflammatory mediators, including inducible nitric oxide synthase (iNOS), cyclooxygenase‐2 (COX‐2), and TNF‐α. However, DPSCs partially suppress p65 expression, thereby reducing TNF‐α production by macrophages. Moreover, IDO expression in DPSCs increases over time in response to LPS or TNF‐α stimulation, suggesting a time‐dependent immunomodulatory response that may contribute to their anti‐inflammatory potential [40]. Irreversible pulpitis tissues showed significant upregulation of TNF‐α and phosphorylated NF‐κB, confirming activation of the NF‐κB‐mediated inflammatory signalling pathway [29]. However, an in vitro study showed that LPS did not alter NF‐κB phosphorylation levels after 20 µg/mL stimulation, even though the expression and secretion of TNF‐α were observed [30]. These findings present emerging evidence that NF‐κB signalling can be spatially and temporally regulated [42].

Recent studies have highlighted the important role of Wnt signalling, a non‐canonical Wnt family member, in mitigating LPS‐induced inflammation and apoptosis in DPCs [43]. Although Wnt signalling is not a direct cellular response to LPS stimulation, it is discussed because its regulatory influence on cytokine production may intersect with LPS‐induced inflammatory pathways, providing relevant mechanistic context. Overexpression of Wnt4 has been shown to suppress the phosphorylation of IKK2, IκBα, and p65, thereby inhibiting the NF‐κB pathway and reducing the expression of IL‐6, IL‐8, TNF‐α and IL‐1β. This suggests that activation of Wnt signalling may serve as a protective mechanism, promoting pulp cell survival and modulating the inflammatory cascade. LPS stimulation (10 µg/mL, 24 h) significantly upregulated the expression of proinflammatory cytokines (IL‐6, IL‐8, TNF‐α, and IL‐1β) and apoptosis‐related proteins (Bax and cleaved caspase‐3), while downregulating anti‐apoptotic Bcl‐2 and Wnt4 expression in hDPCs [43]. In contrast, Wnt inhibition with the chemical inhibitor IWP‐2 in hDPSCs reduced inflammatory gene expression, including *TNFA*, *IL1B*, *IL6* and *IFNG* [5]. Further, IWP‐2 treatment attenuated LPS‐induced inflammatory gene expression via p38 MAPK and PI3K pathways [5]. IWP‐2 inhibits the transportation of Wnt proteins, hence inhibiting both canonical and non‐canonical Wnt pathways. These contradictory findings may reflect differences in cell type, the specific Wnt ligands involved, the dominance of downstream signalling pathways, or context‐dependent responses to LPS. Therefore, further investigation is needed to clarify how distinct branches of the Wnt pathway modulate inflammation in each pulp cell population before definitive conclusions can be drawn.

LPS also regulates non‐coding RNAs. For instance, the long non‐coding RNA (lncRNA) maternally expressed gene 3 (MEG3) is upregulated in both inflamed dental pulp and LPS‐treated cells. Knockdown of MEG3 suppresses the expression of pro‐inflammatory proteins, potentially by disrupting the p38/MAPK pathway. Additionally, MEG3 depletion enhances odontogenic differentiation of hDPCs by activating the Wnt/β‐catenin signalling pathway [15]. Furthermore, LPS‐induced inflammation is tightly regulated by microRNAs. Overexpression of microRNA‐21‐5p, for example, dampens NF‐κB activation and pro‐inflammatory cytokine expression. This anti‐inflammatory effect is thought to be mediated by targeting the untranslated region (UTR) of *TRAF6*, a key adaptor protein in the TLR4 signalling pathway [16]. Various LPS types further intensify inflammation: *P. endodontalis* and *F. nucleatum* LPS enhance IL‐1β expression in gingival fibroblasts and monocytes [44, 45], while IL‐1ra inhibits IL‐1β synthesis induced by *F. nucleatum* LPS [45]. *P. intermedia* LPS also upregulates IL‐6, likely via transcriptional activation, in pulp fibroblasts in a time‐ and dose‐dependent fashion [46].

In addition to soluble mediators, LPS also induces the expression of cell adhesion molecules, including VCAM‐1 and ICAM‐1, on the surface of pulp fibroblasts and endothelial cells [47]. These molecules facilitate leukocyte adhesion and transmigration, thereby amplifying the immune response.

#### 3.1.2. Matrix Degradation and Extracellular Matrix Remodelling

LPS stimulation has been shown to increase the expression of MMPs, particularly MMP‐2 and MMP‐9, which are enzymes responsible for collagen and matrix degradation [48–51]. This enzymatic activity, while part of the normal inflammatory resolution process, can also compromise the structural integrity of the pulp tissue and dentine‐pulp interface if left uncontrolled.

Earlier investigations revealed that while IL‐1 and TNF‐α are strong stimulators of MMP expression in several cell types, their short‐term effects (24–48 h) on MMP production by hDPCs are limited. However, extended exposure (up to 16 days) leads to elevated production of MMP‐2 and MMP‐9, as shown through gelatine zymography, indicating a time‐dependent induction of these proteases in hDPCs under inflammatory conditions [50].

More specifically, Kawai et al. [51] demonstrated that in hDPCs derived from deciduous teeth, LPS significantly increases the gene expression and protein production of MMP‐3 (stromelysin‐1), but not MMP‐1, in a dose‐dependent manner. LPS at concentrations of 1 and 5 µg/mL notably enhanced MMP‐3 expression without altering cell proliferation, indicating that the observed effects were not due to changes in cell growth. MMP‐3 plays a central role not only in ECM degradation but also in activating other MMPs, including gelatinases such as MMP‐2 and MMP‐9, which may further amplify the inflammatory response [51].

In contrast, responses to LPS vary across species and cell types. For example, in a comparative study involving clonal rat pulp cells (RPC‐C2A) and hDPCs, cytokines and LPS increased MMP secretion in rat pulp cells, while hDPCs showed minimal changes under similar short‐term conditions. This discrepancy highlights the importance of cell origin, culture duration and stimulus concentration in evaluating MMP response [52].

Collectively, these findings suggest that LPS is a crucial modulator of MMP production in hDPCs, particularly MMP‐3, and may play a pivotal role in the pathogenesis of pulp inflammation and tissue degradation. Understanding these molecular responses provides insights into the progression of pulpitis and informs therapeutic strategies aimed at preserving or regenerating pulp vitality.

### 3.2. Oxidative Stress and Mitochondrial Dysregulation of DPCs and Stem Cells

#### 3.2.1. Induction of Oxidative Stress

LPS‐induced activation of DPCs is associated with increased reactive oxygen species (ROS) production, which contributes to oxidative stress and cellular damage. The accumulation of ROS also facilitates the activation of redox‐sensitive transcription factors, such as NF‐κB, further perpetuating the inflammatory response [53]. High concentrations or prolonged exposure to LPS can also trigger apoptotic pathways in DPCs. A study on hDPCs revealed that treatment with LPS at 20 µg/mL for 24 h significantly elevated mitochondrial ROS (mtROS) at 6 h, indicating acute oxidative stress. This stress was somewhat mitigated by replacing LPS with regular media or supplementing with Biodentine, Mineral Trioxide Aggregate (MTA), or N‐acetyl cysteine (NAC) [39]. Other studies similarly found that LPS‐induced oxidative stress can be reversed, particularly through upregulation of mitochondrial antioxidants, such as superoxide dismutase 2 (SOD2) [54]. A related study investigated the effects of LPS‐induced inflammation on human apical papilla cells (hAPCs), which are closely related to DPCs due to their anatomical continuity at the apical portion of the pulp. This study focused on oxidative stress, mitochondrial dynamics, and inflammation‐related gene expression. Following LPS stimulation at 20 µg/mL, APCs exhibited a significant upregulation of IL‐6 and TNF‐α, along with increased mtROS production. The study also evaluated the protective effects of NAC, which demonstrated potential to mitigate LPS‐induced cellular alterations [55]. Moreover, LPS treatment has been shown to markedly upregulate mitochondrial antioxidant SOD2, suggesting a cellular response to oxidative stress and a potential protective mechanism against mitochondrial dysfunction. Interestingly, this study compared the inflammatory effects of LPS and H_2_O_2_ and found that SOD2 upregulation was observed only in LPS‐treated cells, not in those treated with H_2_O_2_ alone. This finding underscores the distinct oxidative stress profiles induced by the two agents. LPS appears to trigger a more controlled, mitochondria‐targeted oxidative response, which activates compensatory protective mechanisms such as SOD2 upregulation. In contrast, H_2_O_2_, as a non‐specific exogenous oxidant, may induce more acute and widespread oxidative damage without effectively engaging mitochondrial‐specific antioxidant defence [29].

Another study on the inflamed pulpal tissues demonstrated a significant increase in TNF‐α and phosphorylated NF‐κB, confirming activation of the NF‐κB inflammatory signalling pathway. Alongside this, there was a marked elevation in 4‐hydroxynonenal (4HNE), a lipid peroxidation product, indicating high oxidative stress levels. Interestingly, mitochondrial antioxidant SOD2 was also elevated, possibly representing a compensatory response to oxidative stress [29]. Another recent study confirmed a similar observation, demonstrating a marked increase in oxidative stress following LPS induction. This study focused on a chronic pulpitis model by exposing hDPCs to *Escherichia coli*‐derived LPS (0.1–10 µg/mL) over 7 days; prolonged exposure to 1 and 10 µg/mL LPS showed the most pronounced effects [56].

#### 3.2.2. Mitochondrial Dynamics and Apoptosis

LPS exposure disrupted mitochondrial balance. Experimental studies demonstrate upregulation of pro‐apoptotic proteins such as Bax and cleaved caspase‐3, along with a decrease in anti‐apoptotic Bcl‐2 expression following LPS exposure. These changes contribute to programmed cell death, reducing the pulp’s ability to maintain homeostasis and repair [30]. LPS triggers autophagy in hDPCs through the p38/MAPK pathway [57]. This autophagy activation enhances cell viability compromised by LPS by reducing the expression of IL‐18 and Casp1 [58].

LPS exposure disrupted mitochondrial dynamics in DPCs, as evidenced by a decreased Mfn2/Drp1 gene ratio, indicative of enhanced mitochondrial fission. Although LPS exerted minimal direct effects on apoptosis, as shown by relatively unchanged Bcl‐2/Bax expression, treatment with NAC restored mitochondrial balance by increasing the Mfn2/Drp1 ratio, thereby promoting mitochondrial fusion and improving cellular homeostasis [39]. Similar findings were observed in cells derived from the apical papilla, where LPS induced morphological changes consistent with increased mitochondrial fission, again reflected by a reduced Mfn2/Drp1 ratio at 6 h post‐exposure. Short‐term NAC treatment, particularly at 10 min, effectively downregulated IL‐6 and TNF‐α expression while increasing both Mfn2/Drp1 and Bcl‐2/Bax ratios, suggesting attenuation of inflammation, restoration of mitochondrial fusion, and anti‐apoptotic effects. However, prolonged NAC exposure (>48 h) was found to impair cell proliferation, highlighting the importance of optimising its dosage and timing to maximise therapeutic benefits [55].

Vaseenon et al. [29, 30] conducted a comprehensive analysis comparing healthy and inflamed pulp tissues from patients diagnosed with irreversible pulpitis. Their study confirmed that inflammation is associated with elevated TNF‐α levels and NF‐κB pathway activation. These inflammatory mediators trigger oxidative stress, as evidenced by increased levels of 4‐HNE, and initiate apoptotic pathways in dental pulp tissue [29]. Furthermore, their findings revealed significant mitochondrial dysregulation. An upregulation of mitochondrial fission protein Drp1 and downregulation of mitochondrial fusion proteins MFN2 and OPA1 were observed in inflamed pulp tissues, indicating an imbalance in mitochondrial dynamics. This disruption correlates with increased expression of apoptotic markers, including Bax, cytochrome c, and cleaved caspase‐3, confirming that apoptosis, not necroptosis, is the primary mode of cell death in irreversible pulpitis.

### 3.3. Cellular Senescence

Senescence of DPCs is another critical factor that compromises pulp regeneration, particularly under chronic inflammatory or ageing conditions. The senescent dental stem cells not only exhibit reduced differentiation capacity but also secrete harmful factors that can impair neighbouring cells [59]. The role of LPS in promoting cellular senescence has been documented. Repeated exposure to LPS has been shown to trigger premature ageing in DPSCs, but the underlying mechanisms involve different cellular responses. A study highlights the roles of oxidative stress and DNA damage, showing that increased ROS production in response to LPS activates the DNA damage response (DDR) and upregulates p16^INK4A^, ultimately leading to cell‐cycle arrest and senescence [60]. Meanwhile, another study by the same group highlighted a different route, focusing on the TLR4/MyD88‐NF‐κB‐p53/p21 axis, demonstrating that inflammatory signalling through NF‐κB promotes senescence by enhancing p53 and p21 expression [61]. Together, these findings suggest that DPSC senescence under chronic inflammatory conditions is driven by both oxidative stress and inflammatory signalling, reflecting the multifaceted nature of inflammation‐induced ageing in dental stem cells.

A study evaluated whether two types of bacterial LPS derived from *Porphyromonas gingivalis* and *E. coli* affect the expression of senescence‐related genes in hDPSCs after treatment for 6, 24 and 48 h [62]. The results showed a significant time‐dependent increase in the expression of TP53, CDKN1A, CDKN2A and SIRT1 following LPS stimulation. Notably, *P. gingivalis* LPS predominantly elevated both mRNA and protein expression of CDKN1A and SIRT1, while *E. coli* LPS strongly upregulated TP53 and CDKN2A. These findings highlight the role of bacterial LPS in promoting inflammation‐induced senescence, potentially compromising the regenerative potential of DPSCs.

Controlling cellular senescence is crucial for maintaining the regenerative potential of DPSCs, and recent advancements have introduced promising strategies to mitigate senescence under stress or ageing conditions. A study reported that Nesfatin‐1, a secreted protein expressed in the hypothalamic nuclei showing a promising inhibitory property on LPS‐induced inflammation, attenuates LPS‐induced inflammation and senescence in hDPCs by restoring telomerase activity, reducing the expression of p16 and PAI‐1, and upregulating SIRT1, a key regulator of longevity [63]. Notably, silencing SIRT1 abolished Nesfatin‐1’s protective effects, confirming its essential role in the anti‐senescent mechanism. These findings support the use of targeted agents like Nesfatin‐1 and FK866 to counteract LPS‐induced senescence via oxidative and inflammatory pathways. In a most recent study, Saiyasilp et al. developed an *in vitro* ageing model using D‐galactose (D‐gal) in hDPCs and demonstrated that exposure to 10 g/L of D‐gal significantly promoted cellular senescence, as evidenced by increased SA‐β‐gal activity and upregulation of p16 and p21, both key markers of cell cycle arrest. Together, these studies emphasise not only the detrimental effects of inflammation and oxidative stress on pulp cell ageing but also highlight the therapeutic potential of modulating senescence‐related pathways to preserve stem cell function in dental regenerative applications [64].

### 3.4. Odonto/Osteogenic Potential in DPCs/Stem Cells

Multiple studies report that chronic or high‐dose exposure to LPS negatively affects the odontogenic differentiation of DPCs. LPS impairs odontogenic function in DPCs and DPSCs. LPS exposure reduced mineralisation capacity and downregulated key odontogenic markers, including DMP‐1, DSPP and ALP [30, 39]. An in vitro model simulating chronic pulpitis by exposing DPCs to *E. coli*‐derived LPS (0.1–10 µg/mL) over 7 days revealed that prolonged exposure to 1 and 10 µg/mL of LPS significantly downregulated odontogenic markers (*COL1A1*, *ALPL*, *DSPP* and *DMP1*), elevated pro‐inflammatory cytokines (*TNF*, *IL1B*, *IL8* and *IL6*), increased oxidative stress, and severely compromised mineralisation, collectively simulating the odontogenic dysfunction observed in chronic pulpitis [56]. *P. gingivalis* and *E. coli* LPS at 1 and 10 µg/mL suppressed ALP enzymatic activity and mineralisation in DPSCs in dose dose‐dependent manner, corresponding with the reduction of *ALP*, *COL1A1*, *RUNX2*, *OSX*, *OCN* and *DMP1* mRNA expression [65]. Treatment with the TRIF inhibitor rescued the effects of both *P. gingivalis* and *E. coli* LPS on mineralisation reduction in DPSC, confirming the TLR regulatory pathway [65].

Mechanistically, inflammatory conditions impair the odontogenic potential of DPSCs by downregulating Wnt4 expression. In their study, LPS‐treated DPSCs exhibited significantly reduced alkaline phosphatase (ALP) activity and mineralised nodule formation, effects that were reversed by Wnt4 overexpression [66]. The impairment was mediated through activation of the JNK1 signalling pathway, highlighting the Wnt4/JNK axis as a critical regulator in inflammation‐induced suppression of odontogenic differentiation [66]. These findings highlight the role of Wnt4–JNK signalling in supporting DPSCs function under inflammatory conditions. Another report demonstrated that treatment with NAC, MTA, or Biodentine, following LPS exposure, restored both cell viability and mineralised nodule formation. Notably, NAC—alone or combined with MTA—was particularly effective in reducing inflammation and restoring odontogenic potential [39]. Cannabidiol derived from *Cannabis sativa* rescued the LPS‐suppressed odonto/osteogenic differentiation, evidenced by the upregulation of *COL1A1*, *BMP2*, *RUNX2*, *OSX*, *DSPP* and *DMP1*, as well as mineral deposition in LPS‐treated conditions under maintenance in osteogenic medium [67]. This rescue phenomenon occurred through modulation of the Notch signalling pathway, as confirmed by RNA sequencing and pharmacological inhibition assays [67].

Although LPS is often associated with negative effects on DPSCs, some studies have shown that, under certain conditions, it can support differentiation. Exposure to low concentrations of LPS (1 µg/mL) on days 4 and 7 increased the expression of dentine‐related genes, *DSPP* and *DMP1*, in DPSCs. This effect was linked to the activation of the complement receptor C5aR and the p38 MAPK pathway [21]. When either of these pathways was blocked, the increase in gene expression and mineralisation was reduced, suggesting that mild inflammation could help promote dentine repair through specific immune signals [21]. Similarly, DPSCs from teeth with moderate pulpitis or those treated with low levels of LPS had an improved ability to form dentine and bone‐like tissue. A study showed that treatment with LPS at 1 µg/mL for 3 days significantly enhanced ALP activity, mineralisation, and dentinogenic gene expression, suggesting this condition may promote osteo/odontogenic differentiation. This was linked to an increase in autophagy, a natural cellular process that clears damaged components. When autophagy was blocked, the beneficial effects were lost, confirming its role in supporting differentiation during inflammation [68]. Another study also noted that LPS effects are not always consistent. The responses can vary depending on the physiological state of the cells, the amount of LPS used, and the duration of exposure. The study emphasised that TLR4, a key receptor that detects LPS, can lead to either healing or damage, depending on the immune system’s response. Besides triggering inflammation, LPS also affects DPSCs growth and differentiation by interacting with other signalling pathways, such as MAPK, NF‐κB and autophagy [7].

Recent advances have provided new insights into how LPS impairs the osteogenic and odontogenic differentiation potential of hDPSCs, and how various molecular strategies may counteract these effects. Vaseenon et al. [22] investigated the use of myeloid differentiation factor 2 (MD‐2) inhibitors to mitigate LPS‐induced impairment. Pretreatment with MD‐2 inhibitors, MAC28, L6H21, or 2i‐10, not only suppressed inflammatory signalling via TLR4‐MD‐2 downregulation but also restored mineralised nodule formation and the expression of odontogenic genes. Interestingly, MAC28 and L6H21 effectively reinstated DMP‐1, DSPP, and ALP expression, while 2i‐10 selectively restored only DMP‐1, indicating a partial rescue of odontogenic capacity [22]. Another study identified growth differentiation factor 11 (GDF11) as a potent modulator that rescues LPS‐induced suppression of differentiation by activating SIRT3/FOXO3‐mediated mitophagy. GDF11 overexpression promoted ALP activity, mineralisation, and dentine repair both in vitro and in a rat model of acute pulp injury, while simultaneously reducing inflammatory cytokine levels [69].

In alignment with these emerging insights, overexpression of programmed cell death ligand 1 (PD‐L1) significantly inhibited LPS‐induced inflammatory cytokine expression and enhanced osteo/odontogenic differentiation via upregulation of CCCTC‐binding factor (CTCF), identifying the PD‐L1/CTCF axis as a key immunoregulatory mechanism in regenerative settings [70]. Another study further supported this line of research by developing a collagen sponge/self‐assembling peptide nanofiber scaffold (CS/SAPNS) loaded with LPS‐pretreated human umbilical cord mesenchymal stromal cell (hUCMSC)‐derived small extracellular vesicles (sEVs). This composite scaffold promoted mineralisation and angiogenesis in hDPSCs by activating the NF‐κB signalling pathway, confirming the regenerative impact of LPS‐modulated vesicle therapy [71]. Similarly, a study examining drug‐loaded non‐resorbable polymeric nanoparticles (NPs) on LPS‐induced hDPSCs. The study reported a reduction in mineralisation and in the expression of odontogenic markers, such as ALP, Osteonectin and COL1A1, following LPS induction. In contrast, tideglusib‐doped nanoparticles (TDg‐NPs) preserved cytoskeletal integrity and significantly restored calcium deposition [72].

## 4. Integrated Interpretation and Perspectives

**Figure 2:**
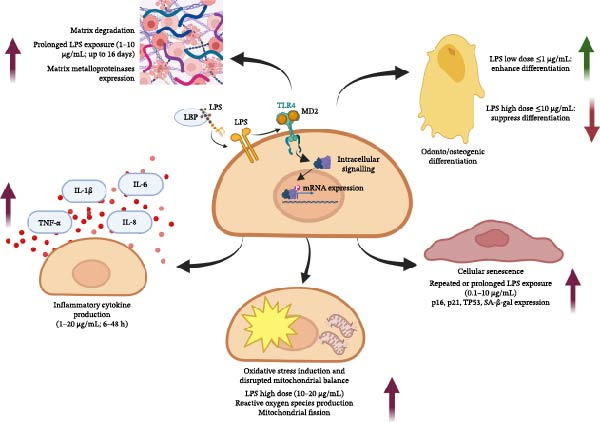
LPS induces a broad range of cellular alterations in dental pulp cells and dental pulp stem cells. Specifically, LPS promotes the production of pro‐inflammatory cytokines, including TNF‐α, IL‐1β, IL‐6 and IL‐8. Prolonged exposure to LPS enhances matrix degradation through the upregulation of matrix metalloproteinases and promotes cellular senescence by modulating the expression of p16, p21, TP53 and SA‐β‐gal. Moreover, high concentrations of LPS induce oxidative stress, disrupt mitochondrial homeostasis, and inhibit odonto‐/osteogenic differentiation. In contrast, low concentrations of LPS have been reported to enhance osteogenic differentiation. Created in BioRender. https://BioRender.com/qdf1m4j.

LPS exerts a wide range of effects on DPCs and DPSCs, influencing inflammation, oxidative balance, mitochondrial activity, cellular ageing and odontogenic potential. Instead of acting through a single pathway, LPS activates several interconnected mechanisms that together influence pulpal inflammation and the tissue’s ability to repair. Overall, a common pattern demonstrated that LPS triggers inflammatory cytokine production and matrix breakdown; continued stimulation disrupts mitochondrial function and increases oxidative stress, which may lead to apoptosis; persistent signalling promotes premature cellular senescence; and these combined effects weaken the cells’ capacity for odonto/osteogenic differentiation (Figure 2). In addition, the different resident cell types could respond differently to stimuli. In this regard, DPCs and DPSCs differ in their stemness and sensitivity to chronic inflammation; many of their downstream responses overlap, particularly in cytokine‐mediated signalling and mitochondrial dysregulation. Understanding these shared and distinct features helps clarify how inflammation progresses and how it might be controlled to preserve pulp vitality.

These molecular insights significantly contribute to clinical relevance. Understanding how LPS‐mediated signalling pathways influence inflammation, oxidative stress, and differentiation capacity not only provides fundamental insight into pulp responses in different clinical environments, such as early carious lesions and advanced pulp infections, but also informs decision‐making for clinical interventions. For instance, cases characterised by low‐grade or transient inflammatory signalling may retain regenerative potential and be suitable for vital pulp therapy or pulp capping, whereas sustained activation of pro‐inflammatory and senescence pathways may indicate irreversible damage, favouring pulpectomy or root canal treatment. Integrating this knowledge into clinical assessment could therefore enhance precision in treatment selection and improve patient outcomes.

Future studies should investigate how LPS dose, exposure duration, and cell type together determine whether the cellular response remains reversible or progresses to irreversible damage. More work is also needed to clarify the roles of major pathways, including TLR4/MD‐2, Wnt, NF‐κB and mitochondrial dynamics, in regulating cytokine production, oxidative stress, senescence, and differentiation. Studies using in vivo or clinically relevant inflammatory models are indeed essential to confirm these mechanisms and to guide the development of targeted strategies, such as pathway inhibitors, senescence‐modulating agents, or pro‐regenerative biomaterials, to maintain pulp vitality and support regenerative endodontic treatment.

## 5. Conclusion

LPS induces a wide spectrum of biological responses in DPCs and DPSCs, including inflammatory activation, oxidative stress, mitochondrial dysfunction, impaired differentiation, and premature senescence. These outcomes are strongly influenced by the dose of LPS. Low concentrations (≤1 µg/mL) could transiently enhance certain defence or reparative activities, while high concentrations (≥10–20 µg/mL) typically drive sustained inflammation and cellular dysfunction. Considering the exposure duration, short‐term stimulation (hours) produces acute signalling changes, and prolonged exposure (days) leads to cumulative stress, apoptosis, or senescence. Understanding how these parameters shape cellular behaviour is essential for interpreting existing findings and for guiding future work aimed at developing strategies to preserve pulp vitality and support regenerative therapies.

## Author Contributions

**Tanida Srisuwan:** conceptualisation, data curation, formal analysis, writing – original draft, writing – review & editing. **Chatvadee Kornsuthisopon:** formal analysis, writing – review & editing. **Nunthawan Nowwarote:** formal analysis, writing – review & editing. **Xiaofei Zhu:** formal Analysis, writing – review & editing. **Waruna Lakmal Dissanayaka:** formal analysis, writing – review & editing. **Thanaphum Osathanon:** project administration, conceptualisation, formal Analysis, writing – review & editing. All authors have contributed significantly and agree with the content of the manuscript.

## Acknowledgments

During the preparation of this work, the authors utilised ChatGPT to enhance readability and language clarity.

## Funding

This study was supported by the Faculty Research Fund (Grant DRF 68_0004), Faculty of Dentistry, Chulalongkorn University.

## Disclosure

After using ChatGPT tool/service, the authors reviewed and edited the content as needed and took full responsibility for the publication’s content. All authors have read and approved the final version of the manuscript. Thanaphum Osathanon had full access to all of the data in this study and takes complete responsibility for the integrity of the data and the accuracy of the data analysis.

## Ethics Statement

The authors declare that ethical approval was not required for this article as it is a review article and does not involve human or animal subjects.

## Conflicts of Interest

The authors declare no conflicts of interest.

## Supporting Information

Additional supporting information can be found online in the Supporting Information section.

## Supporting information


**Supporting Information** Figure S1. Lipopolysaccharide structure (modified from [73]). Created in BioRender. https://BioRender.com/hvb93ef.

## Data Availability

The authors confirm that the data supporting the findings of this study are available within the article and/or its Supporting Information.

## References

[1] Pohl S. , Akamp T. , and Smeda M. , et al.Understanding Dental Pulp Inflammation: From Signaling to Structure, Frontiers in Immunology. (2024) 15, 10.3389/fimmu.2024.1474466, 1474466. 39534600 PMC11554472

[2] Gronthos S. , Mankani M. , Brahim J. , Robey P. G. , and Shi S. , Postnatal Human Dental Pulp Stem Cells (DPSCs) *in vitro* and *in vivo* , Proceedings of the National Academy of Sciences. (2000) 97, no. 25, 13625–13630, 10.1073/pnas.240309797, 2-s2.0-0034610376. PMC17626 11087820

[3] Galler K. M. , Weber M. , Korkmaz Y. , Widbiller M. , and Feuerer M. , Inflammatory Response Mechanisms of the Dentine–Pulp Complex and the Periapical Tissues, International Journal of Molecular Sciences. (2021) 22, no. 3, 10.3390/ijms22031480, 1480. 33540711 PMC7867227

[4] Nakane A. , Yoshida T. , Nakata K. , Horiba N. , and Nakamura H. , Effects of Lipopolysaccharides on Human Dental Pulp Cells, Journal of Endodontics. (1995) 21, no. 3, 128–130, 10.1016/S0099-2399(06)80437-1, 2-s2.0-0029264973. 7561654

[5] Chansaenroj A. , Kornsuthisopon C. , and Suwittayarak R. , et al.IWP-2 Modulates the Immunomodulatory Properties of Human Dental Pulp Stem Cells In Vitro, International Endodontic Journal. (2024) 57, no. 2, 219–236, 10.1111/iej.14001. 37971040

[6] Brodzikowska A. , Ciechanowska M. , Kopka M. , Stachura A. , and Włodarski P. K. , Role of Lipopolysaccharide, Derived From Various Bacterial Species, in Pulpitis—A Systematic Review, Biomolecules. (2022) 12, no. 1, 10.3390/biom12010138, 138. 35053286 PMC8774278

[7] Rodas-Junco B. A. , Hernández-Solís S. E. , Serralta-Interian A. A. , and Rueda-Gordillo F. , Dental Stem Cells and Lipopolysaccharides: A Concise Review, International Journal of Molecular Sciences. (2024) 25, no. 8, 10.3390/ijms25084338, 4338. 38673923 PMC11049850

[8] Fawzy El-Sayed K. M. , Klingebiel P. , and Dörfer C. E. , Toll-Like Receptor Expression Profile of Human Dental Pulp Stem/Progenitor Cells, Journal of Endodontics. (2016) 42, no. 3, 413–417, 10.1016/j.joen.2015.11.014, 2-s2.0-84958885835. 26769027

[9] Farhana A. and Khan Y. S. , Biochemistry, Lipopolysaccharide, 2023, StatPearls Publishing. 32119301

[10] Lamooki S. A. P. , Heris F. S. , Fathi A. , Aminianpour N. , Jandaghian Z. , and Ramandi M. A. , Prevalence and Antimicrobial Resistance of Bacterial Agents Isolated From the Cases of Dental Caries, The International Tinnitus Journal. (2023) 27, no. 2, 211–216, 10.5935/0946-5448.20230032. 38507637

[11] Siqueira J. F.Jr. and Rôças I. N. , Diversity of Endodontic Microbiota Revisited, Journal of Dental Research. (2009) 88, no. 11, 969–981, 10.1177/0022034509346549, 2-s2.0-70350381756. 19828883

[12] Akira S. , Uematsu S. , and Takeuchi O. , Pathogen Recognition and Innate Immunity, Cell. (2006) 124, no. 4, 783–801, 10.1016/j.cell.2006.02.015, 2-s2.0-32944464648. 16497588

[13] He W. , Wang Z. , and Zhou Z. , et al.Lipopolysaccharide Enhances Wnt5a Expression Through Toll-Like Receptor 4, Myeloid Differentiating Factor 88, Phosphatidylinositol 3-OH Kinase/AKT and Nuclear Factor Kappa B Pathways in Human Dental Pulp Stem Cells, Journal of Endodontics. (2014) 40, no. 1, 69–75, 10.1016/j.joen.2013.09.011, 2-s2.0-84890564238. 24331994

[14] Wang X. , Feng Z. , Li Q. , Yi B. , and Xu Q. , DNA Methylcytosine Dioxygenase Ten-Eleven Translocation 2 Enhances Lipopolysaccharide-Induced Cytokine Expression in Human Dental Pulp Cells by Regulating MyD88 Hydroxymethylation, Cell and Tissue Research. (2018) 373, no. 2, 477–485, 10.1007/s00441-018-2826-x, 2-s2.0-85045266607. 29654353

[15] Liu M. , Chen L. , Wu J. , Lin Z. , and Huang S. , Long Noncoding RNA MEG3 Expressed in Human Dental Pulp Regulates LPS-Induced Inflammation and Odontogenic Differentiation in Pulpitis, Experimental Cell Research. (2021) 400, no. 2, 10.1016/j.yexcr.2021.112495, 112495. 33524362

[16] Nara K. , Kawashima N. , and Noda S. , et al.Anti-Inflammatory Roles of microRNA 21 in Lipopolysaccharide-Stimulated Human Dental Pulp Cells, Journal of Cellular Physiology. (2019) 234, no. 11, 21331–21341, 10.1002/jcp.28737, 2-s2.0-85065231559. 31042008

[17] Li D. and Wu M. , Pattern Recognition Receptors in Health and Diseases, Signal Transduction and Targeted Therapy. (2021) 6, no. 1, 10.1038/s41392-021-00687-0, 291. 34344870 PMC8333067

[18] Ciesielska A. , Matyjek M. , and Kwiatkowska K. , TLR4 and CD14 Trafficking and Its Influence on LPS-Induced Pro-Inflammatory Signaling, Cellular and Molecular Life Sciences. (2021) 78, no. 4, 1233–1261, 10.1007/s00018-020-03656-y. 33057840 PMC7904555

[19] Andrukhov O. , Toll-Like Receptors and Dental Mesenchymal Stromal Cells, Frontiers in Oral Health. (2021) 2, 10.3389/froh.2021.648901, 648901. 35048000 PMC8757738

[20] Lee C. C. , Avalos A. M. , and Ploegh H. L. , Accessory Molecules for Toll-Like Receptors and Their Function, Nature Reviews Immunology. (2012) 12, no. 3, 168–179, 10.1038/nri3151, 2-s2.0-84862804952. PMC3677579 22301850

[21] Kim J.-H. , Irfan M. , Hossain M. A. , Shin S. , George A. , and Chung S. , LPS-Induced Inflammation Potentiates Dental Pulp Stem Cell Odontogenic Differentiation Through C5aR and p38, Connective Tissue Research. (2023) 64, no. 5, 505–515, 10.1080/03008207.2023.2218944. 37247252 PMC10524681

[22] Vaseenon S. , Srisuwan T. , Liang G. , Chattipakorn N. , and Chattipakorn S. C. , Myeloid Differentiation Factor 2 Inhibitors Exert Protective Effects on Lipopolysaccharides-Treated Human Dental Pulp Cells via Suppression of Toll-Like Receptor 4-Mediated Signaling, Journal of Dental Sciences. (2024) 19, no. 1, 220–230, 10.1016/j.jds.2023.04.024. 38303896 PMC10829556

[23] Park B. S. and Lee J.-O. , Recognition of Lipopolysaccharide Pattern by TLR4 Complexes, Experimental & Molecular Medicine. (2013) 45, no. 12, 10.1038/emm.2013.97, 2-s2.0-84896575295, e66. 24310172 PMC3880462

[24] Akira S. and Takeda K. , Toll-Like Receptor Signalling, Nature Reviews Immunology. (2004) 4, no. 7, 499–511, 10.1038/nri1391, 2-s2.0-3142724031. 15229469

[25] Lu Y.-C. , Yeh W.-C. , and Ohashi P. S. , LPS/TLR4 Signal Transduction Pathway, Cytokine. (2008) 42, no. 2, 145–151, 10.1016/j.cyto.2008.01.006, 2-s2.0-43049179999. 18304834

[26] Kawai T. , Ikegawa M. , Ori D. , and Akira S. , Decoding Toll-Like Receptors: Recent Insights and Perspectives in Innate Immunity, Immunity. (2024) 57, no. 4, 649–673, 10.1016/j.immuni.2024.03.004. 38599164

[27] Liu Y. , Gao Y. , and Zhan X. , et al.TLR4 Activation by Lipopolysaccharide and *Streptococcus mutans* Induces Differential Regulation of Proliferation and Migration in Human Dental Pulp Stem Cells, Journal of Endodontics. (2014) 40, no. 9, 1375–1381, 10.1016/j.joen.2014.03.015, 2-s2.0-85027947344. 25146018

[28] He W. , Qu T. , and Yu Q. , et al.LPS Induces IL-8 Expression Through TLR4, MyD88, NF-Kappa B and MAPK Pathways in Human Dental Pulp Stem Cells, International Endodontic Journal. (2013) 46, no. 2, 128–136, 10.1111/j.1365-2591.2012.02096.x, 2-s2.0-84872201380. 22788664

[29] Vaseenon S. , Weekate K. , Srisuwan T. , Chattipakorn N. , and Chattipakorn S. , Observation of Inflammation, Oxidative Stress, Mitochondrial Dynamics, and Apoptosis in Dental Pulp Following a Diagnosis of Irreversible Pulpitis, European Endodontic Journal. (2023) 8, no. 2, 148–155, 10.14744/eej.2022.74745. 37010199 PMC10098433

[30] Vaseenon S. , Srisuwan T. , Chattipakorn N. , and Chattipakorn S. C. , Lipopolysaccharides and Hydrogen Peroxide Induce Contrasting Pathological Conditions in Dental Pulpal Cells, International Endodontic Journal. (2023) 56, no. 2, 179–192, 10.1111/iej.13853. 36269677

[31] Kong Q. , Liu L. , Huang Y. , Zhang F. , Wei X. , and Ling J. , The Effect of Octamer-Binding Transcription Factor 4B1 on microRNA Signals in Human Dental Pulp Cells With Inflammatory Response, Journal of Endodontics. (2014) 40, no. 1, 101–108, 10.1016/j.joen.2013.09.030, 2-s2.0-84890559282. 24331999

[32] Mo Z. , Li Q. , Cai L. , Zhan M. , and Xu Q. , The Effect of DNA Methylation on the miRNA Expression Pattern in Lipopolysaccharide-Induced Inflammatory Responses in Human Dental Pulp Cells, Molecular Immunology. (2019) 111, 11–18, 10.1016/j.molimm.2019.03.012, 2-s2.0-85063615501. 30952010

[33] Soe Z. C. , Nan D. N. , and Wahyudi R. , et al.Asiaticoside-Loaded Nanosponges Hydrogel Has an Anti-Inflammatory Effect and Promotes Human Dental Pulp Regeneration, Journal of Endodontics. (2025) 51, no. 7, 931–938, 10.1016/j.joen.2025.04.004. 40246141

[34] Wahyudi R. , Seang S. , Everts V. , Osathanon T. , and Limjeerajarus C. N. , Anti-Inflammatory Effects of the Prostacyclin Analogue Iloprost in an In Vitro Model of Inflamed Human Dental Pulp Cells, Australian Endodontic Journal. (2023) 49, no. S1, 330–338, 10.1111/aej.12736. 36723392

[35] Sugiuchi A. , Sano Y. , Furusawa M. , Abe S. , and Muramatsu T. , Human Dental Pulp Cells Express Cellular Markers for Inflammation and Hard Tissue Formation in Response to Bacterial Information, Journal of Endodontics. (2018) 44, no. 6, 992–996, 10.1016/j.joen.2018.02.022, 2-s2.0-85045556360. 29680724

[36] Wang Y. Y. , Zhu N. X. , Zhao Y. M. , Ge L. H. , and Qin M. , Mineralisation Influence of Betamethasone on Lipopolysaccharide-Stimulated Dental Pulp Cells, The Chinese Journal of Dental Research. (2019) 22, no. 2, 123–129, 10.3290/j.cjdr.a42516, 2-s2.0-85067436187. 31172140

[37] Bindal P. , Ramasamy T. S. , Kasim N. H. A. , Gnanasegaran N. , and Chai W. L. , Immune Responses of Human Dental Pulp Stem Cells in Lipopolysaccharide-Induced Microenvironment, Cell Biology International. (2018) 42, no. 7, 832–840, 10.1002/cbin.10938, 2-s2.0-85045842579. 29363846

[38] Widbiller M. , Eidt A. , and Wölflick M. , et al.Interactive Effects of LPS and Dentine Matrix Proteins on Human Dental Pulp Stem Cells, International Endodontic Journal. (2018) 51, no. 8, 877–888, 10.1111/iej.12897, 2-s2.0-85042109063. 29377169

[39] Weekate K. , Chuenjitkuntaworn B. , and Chuveera P. , et al.Alterations of Mitochondrial Dynamics, Inflammation and Mineralization Potential of Lipopolysaccharide-Induced Human Dental Pulp Cells After Exposure to N-Acetyl Cysteine, Biodentine or ProRoot MTA, International Endodontic Journal. (2021) 54, no. 6, 951–965, 10.1111/iej.13484. 33503268

[40] Lee S. , Zhang Q. Z. , Karabucak B. , and Le A. D. , DPSCs From Inflamed Pulp Modulate Macrophage Function via the TNF-α/IDO Axis, Journal of Dental Research. (2016) 95, no. 11, 1274–1281, 10.1177/0022034516657817, 2-s2.0-84988529700. 27384335 PMC5076759

[41] Gopinath V. K. , Mohammad M. G. , and Sheela S. , Immunomodulatory Effect of IL-1RA in LPS-Activated Macrophage/Dental Pulp Stem Cells Co-Culture, International Endodontic Journal. (2023) 56, no. 1, 27–38, 10.1111/iej.13839. 36190353

[42] Almowallad S. , Alqahtani L. S. , and Mobashir M. , NF-kB in Signaling Patterns and Its Temporal Dynamics Encode/Decode Human Diseases, Life. (2022) 12, no. 12, 10.3390/life12122012, 2012. 36556376 PMC9788026

[43] Ni C. , Wu G. , Miao T. , and Xu J. , Wnt4 Prevents Apoptosis and Inflammation of Dental Pulp Cells Induced by LPS by Inhibiting the IKK/NF-κB Pathway, Experimental and Therapeutic Medicine. (2022) 25, no. 2, 10.3892/etm.2022.11774, 75. 36684653 PMC9842946

[44] Hosoya S. and Matsushima K. , Stimulation of Interleukin-1β Production of Human Dental Pulp Cells by Porphyromonas Endodontalis Lipopolysaccharide, Journal of Endodontics. (1997) 23, no. 1, 39–42, 10.1016/S0099-2399(97)80205-1, 2-s2.0-0030622521. 9594744

[45] Lu H. X. , Xiao M. Z. , and Niu Z. Y. , et al.Effect of IL-1ra on Human Dental Pulp Cells and Pulpal Inflammation, International Endodontic Journal. (2002) 35, no. 10, 807–811, 10.1046/j.1365-2591.2002.00542.x, 2-s2.0-0036777388. 12406373

[46] Tokuda M. , Sakuta T. , Fushuku A. , Torii M. , and Nagaoka S. , Regulation of Interleukin-6 Expression in Human Dental Pulp Cell Cultures Stimulated with *Prevotella intermedia* Lipopolysaccharide, Journal of Endodontics. (2001) 27, no. 4, 273–277, 10.1097/00004770-200104000-00008, 2-s2.0-0035317549. 11485266

[47] Hong J. H. , Kim M. R. , and Lee B. N. , et al.Anti-Inflammatory and Mineralization Effects of Bromelain on Lipopolysaccharide-Induced Inflammation of Human Dental Pulp Cells, Medicina. (2021) 57, no. 6, 10.3390/medicina57060591, 591. 34201357 PMC8227231

[48] Panagakos F. S. , O’Boskey J. F.Jr., and Rodriguez E. , Regulation of Pulp Cell Matrix Metalloproteinase Production by Cytokines and Lipopolysaccharides, Journal of Endodontics. (1996) 22, no. 7, 358–361, 10.1016/S0099-2399(96)80218-4, 2-s2.0-0030185947. 8935061

[49] Zhang P. , Cui Z. , and Li S. , The Protective Effects of S14G-Humanin (HNG) Against Lipopolysaccharide (LPS)- Induced Inflammatory Response in Human Dental Pulp Cells (hDPCs) Mediated by the TLR4/MyD88/NF-κB Pathway, Bioengineered. (2021) 12, no. 1, 7552–7562, 10.1080/21655979.2021.1979914. 34605740 PMC8806744

[50] O’Boskey F. J.Jr. and Panagakos F. S. , Cytokines Stimulate Matrix Metalloproteinase Production by Human Pulp Cells during Long-Term Culture, Journal of Endodontics. (1998) 24, no. 1, 7–10, 10.1016/S0099-2399(98)80203-3, 2-s2.0-0031612945. 9487857

[51] Kawai S. , Harada K. , Daito K. , Arita K. , and Ohura K. , TNF-α and LPS Enhance MMP Production in Human Dental Pulp Cells of Deciduous Teeth, Journal of Hard Tissue Biology. (2012) 21, no. 2, 151–156, 10.2485/jhtb.21.151, 2-s2.0-84862258160.

[52] Kermeoğlu F. , Sayıner S. , Şehirli A. Ö. , Savtekin G. , and Aksoy U. , Does α-Lipoic Acid Therapeutically Effective Against Experimentally Induced-Acute Pulpitis in Rats?, Australian Endodontic Journal. (2023) 49, no. 1, 87–91, 10.1111/aej.12618. 35290687

[53] Mittal M. , Siddiqui M. R. , Tran K. , Reddy S. P. , and Malik A. B. , Reactive Oxygen Species in Inflammation and Tissue Injury, Antioxidants & Redox Signaling. (2014) 20, no. 7, 1126–1167, 10.1089/ars.2012.5149, 2-s2.0-84894073629. 23991888 PMC3929010

[54] Widdrington J. D. , Gomez-Duran A. , and Pyle A. , et al.Exposure of Monocytic Cells to Lipopolysaccharide Induces Coordinated Endotoxin Tolerance, Mitochondrial Biogenesis, Mitophagy, and Antioxidant Defenses, Frontiers in Immunology. (2018) 9, 10.3389/fimmu.2018.02217, 2-s2.0-85054898600, 2217. 30319656 PMC6170658

[55] Jariyamana N. , Chuveera P. , and Dewi A. , et al.Effects of N-Acetyl Cysteine on Mitochondrial ROS, Mitochondrial Dynamics, and Inflammation on Lipopolysaccharide-Treated Human Apical Papilla Cells, Clinical Oral Investigations. (2021) 25, no. 6, 3919–3928, 10.1007/s00784-020-03721-7. 33404763

[56] Soares I. P. M. , Anselmi C. , and Pires M. L. B. A. , et al.Chronic Exposure to Lipopolysaccharides as an In Vitro Model to Simulate the Impaired Odontogenic Potential of Dental Pulp Cells Under Pulpitis Conditions, Journal of Applied Oral Science. (2023) 31, 10.1590/1678-7757-2023-0032, e20230032. 37493701 PMC10382076

[57] Huang Y. , Li X. , Liu Y. , Gong Q. , Tian J. , and Jiang H. , LPS-Induced Autophagy in Human Dental Pulp Cells is Associated With p38, Journal of Molecular Histology. (2021) 52, no. 5, 919–928, 10.1007/s10735-021-10004-2. 34309809

[58] Gao Y. , You X. , and Liu Y. , et al.Induction of Autophagy Protects Human Dental Pulp Cells From Lipopolysaccharide-Induced Pyroptotic Cell Death, Experimental and Therapeutic Medicine. (2020) 19, no. 3, 2202–2210, 10.3892/etm.2020.8475. 32104285 PMC7027320

[59] Morsczeck C. , Cellular Senescence in Dental Pulp Stem Cells, Archives of Oral Biology. (2019) 99, 150–155, 10.1016/j.archoralbio.2019.01.012, 2-s2.0-85060344155. 30685471

[60] Feng X. , Feng G. , and Xing J. , et al.Repeated Lipopolysaccharide Stimulation Promotes Cellular Senescence in Human Dental Pulp Stem Cells (DPSCs), Cell and Tissue Research. (2014) 356, no. 2, 369–380, 10.1007/s00441-014-1799-7, 2-s2.0-84902251582. 24676500

[61] Feng G. , Zheng K. , and Cao T. , et al.Repeated Stimulation by LPS Promotes the Senescence of DPSCs via TLR4/MyD88-NF-κB-p53/p21 Signaling, Cytotechnology. (2018) 70, no. 3, 1023–1035, 10.1007/s10616-017-0180-6, 2-s2.0-85042534649. 29480340 PMC6021280

[62] Sattari M. , Masoudnia M. , and Mashayekhi K. , et al.Evaluating the Effect of LPS From Periodontal Pathogenic Bacteria on the Expression of Senescence-Related Genes in Human Dental Pulp Stem Cells, Journal of Cellular and Molecular Medicine. (2022) 26, no. 22, 5647–5656, 10.1111/jcmm.17594. 36259309 PMC9667521

[63] Zhang L. , Pang B. , Wang R. , Yang B. , and Jia X. , Nesfatin-1 Attenuated Lipopolysaccharide-Induced Inflammatory Response and Senescence in Human Dental Pulp Cells, Heliyon. (2024) 10, no. 12, 10.1016/j.heliyon.2024.e32108, e32108. 38975143 PMC11226773

[64] Saiyasilp S. , Vaseenon S. , Srisuwan T. , and Chuveera P. , Effects of D-Galactose Induction on Aging Characteristics of the Human Dental Pulp Cell Culture Model: An In Vitro Study, European Endodontic Journal. (2025) 10, no. 2, 142–150, 10.14744/eej.2024.15010. 40143562 PMC11971696

[65] Bulanawichit W. , Sinsareekul C. , and Kornsuthisopon C. , et al.Toll-Like Receptor and C-Type Lectin Receptor Agonists Attenuate Osteogenic Differentiation in Human Dental Pulp Stem Cells, BMC Oral Health. (2024) 24, no. 1, 10.1186/s12903-024-03894-7, 148. 38297241 PMC10832253

[66] Zhong T. Y. , Zhang Z. C. , and Gao Y. N. , et al.Loss of Wnt4 Expression Inhibits the Odontogenic Potential of Dental Pulp Stem Cells through JNK Signaling in Pulpitis, American Journal of Translational Research. (2019) 11, no. 3, 1819–1826. 30972205 PMC6456534

[67] Kornsuthisopon C. , Chansaenroj A. , and Suwittayarak R. , et al.Cannabidiol Alleviates LPS-Inhibited Odonto/Osteogenic Differentiation in Human Dental Pulp Stem Cells In Vitro, International Endodontic Journal. (2025) 58, no. 3, 449–466, 10.1111/iej.14183. 39697062

[68] Yu S. , Liu X.-M. , and Liu Y. , et al.Inflammatory Microenvironment of Moderate Pulpitis Enhances the Osteo-/Odontogenic Potential of Dental Pulp Stem Cells by Autophagy, International Endodontic Journal. (2024) 57, no. 10, 1465–1477, 10.1111/iej.14108. 39031653

[69] Deng M. , Tang R. , Xu Y. , Xu Y. , and Chen L. , GDF11 Promotes Osteogenic/Odontogenic Differentiation of Dental Pulp Stem Cells to Accelerate Dentin Restoration via Modulating SIRT3/FOXO3-Mediated Mitophagy, International Immunopharmacology. (2024) 142, 10.1016/j.intimp.2024.113092, 113092. 39317051

[70] Zhu Y. , Chen M. , Liu F. , Li B. , and He Y. , Overexpression of Programmed Cell Death Ligand 1 Reduces LPS-Induced Inflammatory Cytokine Upregulation and Enhances Osteo/Odontogenic-Differentiation of Human Dental Pulp Stem Cells via Upregulation of CCCTC-Binding Factor, Archives of Oral Biology. (2024) 165, 10.1016/j.archoralbio.2024.106031, 106031. 38905870

[71] Zeng J. , Deng H. , Li Q. , Kang J. , and Wu Y. , Scaffold Loaded LPS-hUCMSC-sEVs Promote Osteo/Odontogenic Differentiation and Angiogenic Potential of hDPSCs, Tissue and Cell. (2024) 91, 10.1016/j.tice.2024.102549, 102549. 39226663

[72] Osorio R. , Rodríguez-Lozano F. J. , and Toledano M. , et al.Mitigating Lipopolysaccharide-Induced Impairment in Human Dental Pulp Stem Cells With Tideglusib-Doped Nanoparticles: Enhancing Osteogenic Differentiation and Mineralization, Dental Materials. (2024) 40, no. 10, 1591–1601, 10.1016/j.dental.2024.07.012. 39068091

[73] Serrato R. V. , Lipopolysaccharides in Diazotrophic Bacteria, Frontiers in Cellular and Infection Microbiology. (2014) 4, 10.3389/fcimb.2014.00119, 2-s2.0-84907945657, 119. 25232535 PMC4153317

