# Supplementary figures and images for: Lipopolysaccharides Modulate Cellular Responses in Dental Pulp Cells
Source: Int J Dent. 2026 Apr 23;2026:2049490. doi: 10.1155/ijod/2049490 (PMC13106950; doi:10.1155/ijod/2049490)

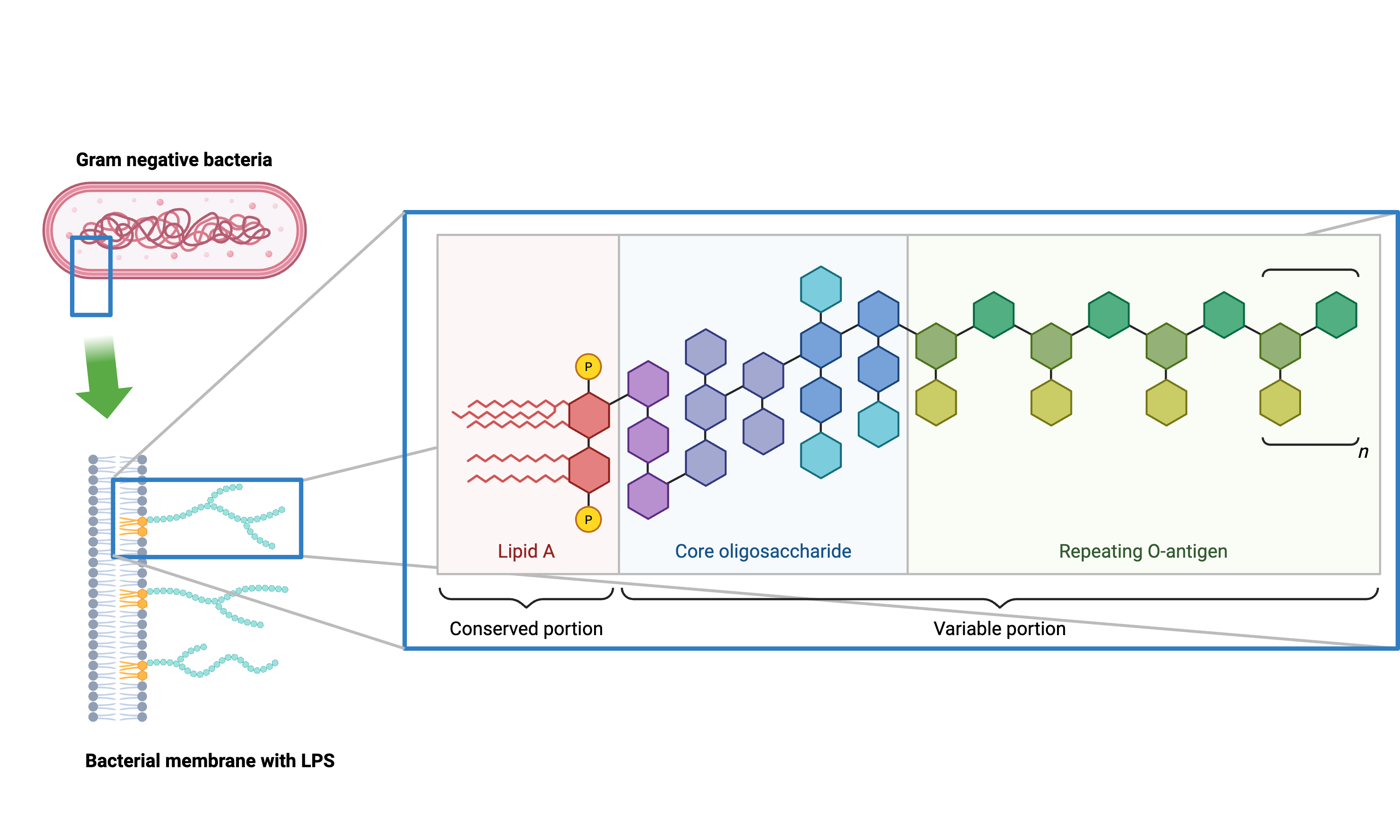

Supplement: Supplementary file 1 — Supporting Information Figure S1. Lipopolysaccharide structure (modified from [73]). Created in BioRender. https://BioRender.com/hvb93ef. [file IJOD-2026-2049490-s001.png]
